# A Cross-Sectional Study of the Prevalence and Determinants of Common Mental Health Problems in Primary Care in Switzerland

**DOI:** 10.3389/ijph.2023.1606368

**Published:** 2023-12-15

**Authors:** Juliane Messer, Konstantinos Tzartzas, Régis Marion-Veyron, Christine Cohidon

**Affiliations:** ^1^ Department of Family Medicine, University Center of General Medicine and Public Health, Lausanne, Switzerland; ^2^ Faculty of Biology and Medicine, University of Lausanne, Lausanne, Switzerland; ^3^ Department of Ambulatory Care and Community Medicine, University Center of General Medicine and Public Health, Lausanne, Switzerland

**Keywords:** mental health, general practitioners, anxiety, depression, primary care

## Abstract

**Objective:** This study investigated the prevalence of the most common mental health symptoms in a large primary care patient population and characterized their determinants.

**Methods:** Data came from a 2015–16 cross-sectional study of a primary care population in Switzerland. An investigator presented the study to patients in waiting rooms, and 1,103 completed a tablet-based questionnaire measuring stress in daily life, sleep disorders and anxiety and depressive symptoms. Diagnoses and treatments were recorded.

**Results:** Moderate-to-high anxiety and depressive symptoms concerned 7.7% of patients; 27.6% felt stressed at least once a week; 17.2% had severe sleep disorders. Sociodemographic determinants were associated with psychiatric symptoms: female sex, young age, and frequency of consultations with a GP. Participants taking psychotropics had high levels of mental distress.

**Conclusion:** Even though most patients were regularly monitored by their GP, a significant number of mental health problems were found. GPs should be provided with concrete tools to manage these patients better. Collaboration with mental health specialists should be encouraged in primary care settings.

## Introduction

Mental health problems put significant burdens on patients, families, and healthcare systems more broadly. In Europe in 2011, the most prevalent 12 months disorders were anxiety disorders (14.0%), mood disorders (7.8%), especially major depression (6.9%), and insomnia (7.0%) [1]. A 2022 World Health Organization report ranked depressive disorders as the second largest contributor to non-fatal declining health worldwide, responsible for 5.6% of all Years Lived with Disability (YLD); anxiety disorders were the sixth-largest contributor (3.4% of all YLD) [2]. The 2017 Global Burden of Disease study found that 13.9% of all the Disability-Adjusted Life Years lost in Switzerland were caused by mental health problems [3, 4].

In primary care contexts, which is mainly represented by general practitioners (GPs) practices, many of these disorders are not properly diagnosed or are poorly treated because GPs sometimes feel that they lack the skills to treat mental health problems and cannot offer their patients enough consultation time for these problems [5–10]. Additionally, despite some GPs not having the proper screening tools to detect mental health problems [7], they are often the first healthcare professionals to be in contact with these patients, before any specialists [7, 11, 12]. A prevalence of 25%–60% of mental disorders in primary care medicine has been reported worldwide [13, 14]. In a study conducted in 2008–2009 in Switzerland, GPs estimated that 30% of their patients presented with symptoms of depression [15]. And they often have to take care of patients with mental health problems over the long term [7].

In 2020, the Swiss Health Observatory used Swiss Health Survey and other national survey data to reveal that 15.1% of Switzerland’s population reported having moderate-to-severe psychological distress. This indicator was measured using the Mental Health Inventory-5, which investigates the frequency with which patients have felt three negative emotional states (nervousness, bad mood, discouragement) and two positive emotional states (calm or peacefulness, feelings of happiness) in the past 4 weeks [16]. Scores do not necessarily equate to a medical diagnosis, but higher levels of self-reported psychological distress are linked to increased probabilities of presenting with a mental disorder. More than a third of respondents, for example, reported having mild-to-severe symptoms of depression (3% severe; 6% moderate; 26% mild) [3].

However, statistics are missing about the prevalence of GP consultations for common mental health problems. The data in most prevalence studies are derived from surveys conducted among the general population, and the links with primary care are not well explored. It means that the use of primary care in these surveys is not investigated. The present study used data collected directly from patients monitored by a GP. This study’s findings could, therefore, help GPs anticipate and target screenings and assist other stakeholders in proposing more optimal public health policies.

We know that there are many social determinants of mental health, which is why we choose different variables likely to be associated with mental health disorders in our study. The indicators we use in the analysis models have not been chosen randomly, but based on the literature. It has been shown, for example, that a person’s socio-economic level can influence his or her mental health [17–19]. Demographic indicators such as gender or age are also important to take into account, in order to stratify the population studied and according to the results [20, 21]. This makes it possible to better target certain populations affected by one disorder or another in prevention initiatives for example. Certain health behaviours or lifestyle habits may also be associated with poor or good mental health, such as alcohol consumption, smoking or physical activity [22]. In the spirit of holistic care, we do not want to focus only on the medical aspect and symptomatology. Looking at the patient’s lifestyle and environment seems important to better understand the mental health disorders encountered in family medicine.

The present study’s primary objective was to determine the prevalence of the most common mental health problems (depression, anxiety, sleep disorders, and stress) in a large primary care patient population and characterize their determinants. Its secondary objective was to observe whether mental health problems were associated with patients’ self-reported psychiatric diagnoses and psychotropic treatments.

## Methods

Data came from a survey conducted for the Swiss Primary care Active Monitoring (SPAM-Prev) program on prevention in family medicine in Switzerland [23, 24]. In 2012, the SPAM research network began collecting data for several studies involving family medicine and was constituted of GPs willing to be part of the network.

The present study is a national, cross-sectional survey conducted in 2015–16 by the University Center for Primary Care and Public Health’s Department of Family Medicine (formerly the University Medical Polyclinic) in Lausanne. Its objective was to monitor primary care activities and professionals in Switzerland. We had a 60% participation rate: 170 GPs from the SPAM network participated in this study, representativeness was verified on the basis of sex, gender and region [25]. These GPs asked their patients to participate too. A trained investigator was assigned to each medical practice to administer a questionnaire to up to 10 patients using a tablet computer. A total of 1,157 patients were included (a mean of about 7 patients per GP), and data on sociodemographic variables, patients’ perceptions of their health and their opinions about prevention were investigated. Their use of care, medical history, and treatments were also obtained.

The study was approved by the Human Research Ethics Committee of the Canton of Vaud (N°74/15), and all patients signed a written informed consent form.

### Data

For the present study, we selected nine items from the SPAM questionnaire about mental health:

- The Patient Health Questionnaire-4 (PHQ-4) is a four-item questionnaire on anxiety and depression combining a two-item measure (PHQ-2) of the core criteria for depression and a two-item measure for anxiety (Generalized Anxiety Disorder 2-item or GAD-2), both of which have been independently shown to be good, brief screening tools [26–28]. A PHQ-2 is positive if the score is equal or superior to 3 points, situation in which we consider the patient to have a high probability to have a major depression or another depressive trouble [29]. A GAD-2 is positive if the score is equal or superior to 3 points, situation in which we consider the patient to have a high probability to have one of the following issues: generalized anxiety disorder, panic disorder, social anxiety disorder or posttraumatic stress disorder [28]. The PHQ-4 is used to identify individuals at risk of anxiety and/or depressive disorders, and the score is distributed as follows: 0–2 points: normal risk, 3–5 points: mild risk, 6–8 points: moderate risk, 9–12 points: severe risk [26].
- The frequency of exposure to stress was measured by the question “Do you feel stressed in your daily life?” The three possible answers were “never,” “occasionally,” and “often (1/week) or very often (>1/week)”.
- A sleep quality score was based on four questions: “Generally, do you have difficulties falling asleep?”; “Do you ever wake up during the night and have difficulties falling asleep again?”; “Do you ever wake up too early in the morning without being able to fall asleep again?” and “Generally, do you evaluate your sleep as being regenerative, meaning that it enables you to recover from the fatigue of the day?” ([30–32]). Each positive answer to the first three questions and each negative answer to the last question added one point to the final score, resulting in four categories: 0 = no sleep disorders, 1 = mild sleep disorder, 2 = moderate sleep disorder, and 3 or 4 points = severe sleep disorder.

We also used data on medical diagnoses and treatments self-reported by patients:

- Diagnosed medical conditions or disorders self-reported by patients in answer to the question “Do you have any chronic illnesses? If so, which one?” were coded using the International Classification of Diseases 10th revision (ICD-10). We retained all the self-reported diagnoses concerning mental health (F00–F99 mental health and behavioral disorders).
- Medications were coded using the Anatomical Therapeutic Chemical (ATC) classification system). We retained all the antipsychotics, anxiolytics, hypnotics and sedatives, antidepressants, and psychostimulants (N05 and N06).

### Statistical Analysis

We began by making a descriptive analysis of the participants’ sociodemographic characteristics, their self-reported psychiatric diagnoses and psychotropic treatments and some of their health behaviors. We then described patients’ mental health status based on their self-reported psychiatric diagnoses and psychotropic treatments, the PHQ-4’s items on anxiety and depression, and their self-reported frequency of exposure to stress and the severity of sleep disorders. Finally, the associations between these three main indicators (PHQ-4, exposure to stress and severity of sleep disorders) and patients’ sociodemographic, health-related, and lifestyle-related variables were analyzed using ordered logistic regressions (appropriate model for ordinal categorical data). The sociodemographic variables included were sex, age (studied in a quantitative or categorial mode, depending on the model’s performance), country of birth, family status, educational level (vocational training is equivalent to apprenticeship or high school level, and higher education means a degree superior to high school), and employment status. Health-related variables were perceived health (scored on a scale from 0 to 100), the number of consultations with a GP in the last 12 months, and body mass index (BMI = weight [kg]/height^2^ [m^2^]). Lifestyle-related variables were tobacco use, alcohol consumption (AUDIT-C) [33, 34], cannabis use, playing sports, and eating habits. We performed univariate and multivariate analyses to investigate which factors (independent sociodemographic, health-related, and lifestyle-related variables) were predictive of the three indicators (mental health-related dependent variables). In our univariate analyses, variables associated with a *p*-value of ≤0.2 were selected to build three final multivariate models using manual stepwise selection (removal of the least significant variable at each step). Statistical analyses were performed using STATA software (Version 14.2).

## Results

The sociodemographic, health, and lifestyle characteristics of the 1,103 patients included in the study are described in Table 1. The difference from the original number of participants was due to 54 patients for whom we only had information about treatment and diagnosis, so they were excluded from the analyses. Mean patient age was 58 years old, 56.6% were women, 40.3% were in employment, and 44.2% were retired. Participants’ mean perceived health score was 70.6, 2.4% reported one or more psychiatric diagnoses, and 6% were taking a psychotropic treatment. The distribution of the psychotropic drugs taken is shown in Table 1. Of the patients diagnosed with a psychiatric disorder, 32.1% were taking a psychotropic treatment.

**TABLE 1:** Sample characteristics (Lausanne, Switzerland. 2021).

		n (N = 1,103)	%
Sex			
	Male	479	43.5
	Female	623	56.5
Age			
	15–34	156	14.4
	35–49	179	16.5
	50–65	266	24.5
	>65	484	44.6
Country of birth			
	Switzerland	830	75.3
	Other	273	24.7
Employment status			
	Employed	424	40.3
	Retired	465	44.2
	Student/Apprentice	41	3.9
	Unemployed/Inactive	121	11.5
Educational level			
	Obligatory schooling or less	190	18.1
	Vocational training	568	53.9
	Higher education	295	28.0
Family status			
	Couple (with children)	232	21.1
	Couple (no children)	455	41.3
	Living alone with children	46	4.2
	With parents	62	5.6
	Living alone	306	27.8
Perceived health score (scale from 0 to 100)			
	0–55	281	25.6
	56–80	433	39.4
	81–90	229	20.9
	91–100	155	14.1
Body Mass Index			
	Underweight (<18.5 kg/m^2^)	25	2.6
	Normal (>18.5<25 kg/m^2^)	422	43.2
	Overweight (>25<30 kg/m^2^)	308	31.5
	Obese (>30 kg/m^2^)	222	22.7
Self-reported psychiatric diagnosis			
	Yes	27	2.4
	No	1,073	97.6
Psychotropic treatment			
	Yes	66	6.0
	Type of medication^a^		
	Antidepressant	45	4.1
	Antipsychotic	7	0.6
	Anxiolytic	22	2.0
	Others	4	0.4
	No	1,034	94.0
Consultations with a GP in the last 12 months			
	0	78	7.01
	1–2	290	26.3
	3–5	343	31.1
	>6	392	35.5
Tobacco use			
	Yes	235	21.4
	No	865	78.6
Hazardous alcohol consumption (audit-c)			
	Yes	391	35.5
	No	711	64.5
Cannabis use (last 30 days)			
	Yes	51	4.70
	No	1,043	95.3
Sports frequency			
	Very often (>1/week)	370	33.8
	Often (1/week)	207	18.9
	Occasionally	218	19.9
	Never	300	27.4
Balanced diet			
	Yes	902	82.7
	No	153	14.0
	Does not know	36	3.3

^a^ Some patients were undergoing more than one psychotropic treatment; thus, we have a sum greater than 6%.

The largest group (43.6%) of patients reported never being stressed during their daily life, 28.8% were occasionally stressed, and 27.6% were often (1/week) or very often (>1/week) stressed. Concerning the PHQ-4, 19.1% showed mild symptoms of anxiety and depression, and 7.7% showed a moderate-to-high score: 9.7% of patients had a positive GAD-2 score, and 10.5% had a positive PHQ-2. Regarding sleep disorders, 28.7% of patients reported having general difficulties falling asleep, 36.6% sometimes woke up during the night and had difficulties falling asleep again, 30.2% sometimes awoke too early in the morning without being able to fall asleep again, and 21.5% estimated that they did not get regenerative sleep. In total, more than half (58.1%) had one or more of these sleep issues. Overall, patients with a self-reported psychiatric diagnosis felt more frequently exposed to stress, had a higher PHQ-4 score, and had more severe sleep disorders. The same tendency was observed among participants taking psychotropic drugs (Table 2).

**TABLE 2:** Mental health-related variables: symptom, self-reported diagnosis, and psychotropic treatment frequencies (%) (Lausanne, Switzerland. 2021).

		All patients N (%)	Patients with a psychiatric diagnosis	Patients undergoing a psychotropic treatment
Patient Health Questionnaire-4	Normal	758 (73.2%)	10 (37%)	25 (38.5%)
	Mild	198 (19.1%)	7 (25.9%)	23 (35.4%)
	Moderate/Severe	80 (7.7%)	10 (37%)	17 (26.1%)
Stress frequency	Never	481 (43.6%)	5 (18.5%)	23 (34.9%)
	Occasionally	318 (28.8%)	13 (48.2%)	22 (33.3%)
	Often/very often (>1/week)	304 (27.6%)	9 (33.3%)	21 (31.8%)
Sleep disorders	No	432 (41.9%)	4 (14.8%)	18 (28.1%)
	Mild	243 (23.5%)	10 (37%)	14 (21.9%)
	Moderate	188 (17.4%)	4 (14.8%)	15 (23.4%)
	Severe	177 (17.2%)	9 (33.4%)	17 (26.6%)

The variables associated with the frequency of exposure to stress are provided in Table 3. In the final multivariate analysis model, females were twice as likely to be stressed as males (OR = 2.09 [1.63–2.69]). Less frequent stress was associated with older age, especially among participants older than 65 (OR = 0.42 [0.22–0.79]), a high level of perceived health (OR = 0.995 [0.98–1.00], and a balanced diet (OR = 0.55 [0.38–0.78]). A high frequency of exposure to stress was associated with cannabis use (OR = 2.16 [1.18–3.96]), physical inactivity (OR = 1.47 [1.06–2.03]), regular sport practice (once a week) (OR = 1.44 [1.02–2.03]), and even more to an occasional sport practice (OR = 1.64 [1.16–2.32]) compared to a very frequent (more than once a week) practice. A lower frequency of exposure to stress was associated with people living in couple without children (OR = 0.68 [0.49–0.95]), alone with children (OR = 0.52 [0.28–0.97]) or alone without children (OR = 0.58 [0.41–0.83]) compared to couples with children or people living with their parents.

**TABLE 3:** Patient characteristics associated with perceived frequency of stress (ordered logistic regression) (Lausanne, Switzerland. 2021).

				Univariate analyses		Multivariate analyses	
		n	%	OR	95% CI	OR	95% CI
Sex (ref: Male)							
	Female	623	56.5	**1.81**	**1.44–2.26**	**2.09**	**1.63–2.69**
Age (ref: 15–34)							
	35–49	179	15.7	0.82	0.55–1.21	0.76	0.48–1.20
	50–65	266	23.4	**0.59**	**0.41–0.85**	0.65	0.42–1.01
	>65	538	47.2	**0.31**	**0.22–0.44**	**0.42**	**0.22–0.79**
Country of birth (ref: Switzerland)							
	Other	273	24.7	**1.36**	**1.06–1.76**	-	-
Employment status (ref: Employed)							
	Retired	465	44.24	**0.28**	**0.22–0.37**	**0.56**	**0.32–0.96**
	Student/Apprentice	41	3.9	1.01	0.56–1.85	0.65	0.32–1.34
	Unemployed/Inactive	121	11.5	1.11	0.77–1.61	0.99	0.67–1.47
Educational level (ref: Obligatory schooling or less)							
	Vocational training	568	53.9	1.11	0.81–1.51	-	-
	Higher education	295	28	**1.44**	**1.03–2.03**	-	-
Family status (ref: Couple (with children)/With parents)							
	Couple (no children)	455	41.3	**0.42**	**0.32–0.55**	**0.68**	**0.49–0.95**
	Living alone with children	46	4.2	**0.66**	**0.37–1.16**	**0.52**	**0.28–0.97**
	Living alone	306	27.8	**0.46**	**0.34–0.62**	**0.58**	**0.41–0.83**
Perceived health score (scale from 0 to 100, continuous variable)							
		1,098	100	**0.995**	**0.99–0.999**	**0.995**	**0.98–1.00**
Body Mass Index (ref: Normal and underweight (<25 kg/m^2^))							
	Overweight (>25<30 kg/m^2^)	308	31.5	0.91	0.69–1.19	-	-
	Obese (>30 kg/m^2^)	222	22.7	1.19	0.88–1.61	-	-
Self-reported psychiatric diagnosis (ref: No)							
	Yes	28	2.4	1.79	0.94–3.43	-	-
Psychotropic treatment (ref: No)							
	Yes	68	5.9	1.28	0.82–1.99	-	-
Consultations with a GP in the last 12 months (ref: 0)							
	1–2	290	25.1	1.10	0.69–1.76	-	-
	3–5	343	29.7	0.88	0.55–1.40	-	-
	>6	446	38.5	1.32	0.84–2.08	-	-
Tobacco use (ref: No)							
	Yes	235	21.4	**1.51**	**1.16–1.98**	-	-
Hazardous alcohol consumption (audit-c) (ref: No)							
	Yes	391	35.5	1.21	0.96–1.52	-	-
Cannabis use (last 30 days) (ref: No)							
	Yes	51	4.7	**2.96**	**1.74–5.05**	**2.16**	**1.18–3.96**
Sports frequency (ref: Very often (>1/week))							
	Often (1/week)	207	18.9	**1.35**	**0.99–1.84**	**1.44**	**1.02–2.03**
	Occasionally	218	19.9	**1.78**	**1.30–2.43**	**1.64**	**1.16–2.32**
	Never	300	27.4	1.03	0.77–1.37	**1.47**	**1.06–2.03**
Balanced diet (ref: No)							
	Yes	938	86	**0.42**	**0.31–0.58**	**0.55**	**0.38–0.78**

Results in bold are significant.

In the final multivariate analysis model, a higher PHQ-4 score was associated (but was barely significant) with female sex (OR = 1.31 [0.97–1.77]) and being born outside Switzerland (OR = 1.6 [1.16–2.21]). It was also associated with high numbers of consultations with a GP in the last 12 months (OR = 1.78 [1.14–2.77]), low levels of perceived health (OR = 0.99 [0.98–0.99], physical inactivity (OR = 2.05 [1.38–3.04]), and employment status: unemployed patients (job seekers or those receiving invalidity insurance payments, etc.) were more likely to score high on the PHQ-4 scale (OR = 1.78 [1.14–2.77]) than patients in employment. Lower PHQ-4 scores were associated with older age (OR = 0.98 [0.97–0.99], a continuous variable) and a balanced diet (OR = 0.57 [0.38–0.83]) (Table 4).

**TABLE 4:** Patient characteristics associated with Patient Health Questionnaire-4 score (ordered logistic regression) (Lausanne, Switzerland. 2021).

				Univariate analyses		Multivariate analyses	
		n	%	OR	95% CI	OR	95% CI
Patients		1,157					
Sex (ref: Male)							
	Female	591	57.1	1.39	1.05–1.84	1.31	0.97–1.77
Age (continuous variable)							
		1,019		0.99	0.98–0.99	**0.98**	**0.97–0.99**
Country of birth (ref: Switzerland)							
	Other	255	24.6	**1.72**	**1.27–2.32**	**1.60**	**1.16–2.21**
Employment status (ref: Employed)							
	Retired	442	43.7	0.81	0.60–1.11	1.09	0.66–1.84
	Student/Apprentice	41	4	1.00	0.49–2.06	0.98	0.43–2.26
	Unemployed/Inactive	119	11.8	**2.31**	**1.52–3.5**	**1.78**	**1.14–2.77**
Education level (ref: Obligatory schooling or less)							
	Vocational training	540	53.3	**0.68**	**0.48–0.97**	-	-
	Higher education	288	28.4	**0.61**	**0.41–0.92**	-	-
Family status (ref: Couple with children/With parents)							
	Couple (no children)	421	40.7	0.69	0.50–0.99	0.82	0.55–1.24
	Living alone with children	45	4.4	**1.87**	**1.01–3.46**	**1.98**	**1.02–3.84**
	Living alone	291	28.1	**1.10**	**0.74–1.51**	**1.06**	**0.69–1.62**
Perceived health score (scale from 0 to 100, continuous variable)							
		1,034		**0.98**	**0.98–0.99**	**0.99**	**0.98–0.99**
Body Mass Index (ref: Normal or underweight (<25 kg/m^2^))							
	Overweight (>25 < 30 kg/m^2^)	291	31.7	**1.69**	**1.20–2.37**	-	-
	Obese (>30 kg/m^2^)	211	22.9	**1.61**	**1.11–2.32**	-	-
Self-reported psychiatric diagnosis (ref: No)							
	Yes	27	2.6	**6.1**	**2.91–12.77**	-	-
Psychotropic treatment (ref: No)							
	Yes	65	6.3	**4.97**	**3.08–8.01**	-	-
Consultations with a GP in the last 12 months (ref: 0)							
	1–2	279	26.9	1.43	0.73–2.82	1.43	0.70–2.90
	3–5	328	31.7	1.71	0.88–3.31	1.85	0.92–3.71
	>6	355	34.3	**2.80**	**1.46–5.39**	**2.59**	**1.30–5.18**
Tobacco use (ref: No)							
	Yes	224	21.6	**1.41**	**1.02–1.94**	-	-
Hazardous alcohol consumption (audit-c) (ref: No)							
	Yes	362	35	0.84	0.63–1.12	-	-
Cannabis use (last 30 days) (ref: No)							
	Yes	49	4.7	1.83	1.02–3.26	-	-
Sports frequency (ref: Very often (>1/week))							
	Often (1/week)	199	19.2	1.49	0.99–2.24	**1.67**	**1.08–2.59**
	Occasionally	205	19.8	**1.88**	**1.26–2.80**	**1.58**	**1.04–2.42**
	Never	280	27	**1.29**	**1.60–3.28**	**2.05**	**1.38–3.04**
Balanced diet (ref: No)							
	Yes	888	85.8	**0.44**	**0.31–0.62**	**0.57**	**0.38–0.83**

Results in bold are significant.

In the final multivariate analysis model, a higher risk of reported sleep disorder was associated with female sex (OR = 1.34 [1.06–1.69]) and more consultations with a GP in the last 12 months (OR = 1.69 [1.03–2.78]). Factors associated with fewer sleep disorders were a good perceived health (OR = 0.99 [0.98–0.99]) and educational level: vocational training (OR = 0.72 0.52–0.99) compared to obligatory schooling or less. No associations with age were revealed, but we maintained this variable in the final model to ensure overall coherence (Table 5).

**TABLE 5:** Patient characteristics associated with sleep disorders (ordered logistic regression) (Lausanne, Switzerland. 2021).

				Univariate analyses		Multivariate analyses	
		n	%	OR	95% CI	OR	95% CI
Sex (ref: Male)		1,157					
	Female	578	56.1	**1.33**	**1.06–1.67**	**1.34**	**1.06–1.69**
Age (ref: 15–34)							
	35–49	172	16.9	0.98	0.65–1.46	0.97	0.65–1.47
	50–65	246	24.2	1.29	0.89–1.86	1.22	0.83–1.78
	>65	451	44.4	0.92	0.66–1.30	0.83	0.58–1.18
Country of birth (ref: Switzerland)							
	Other	256	24.8	1.20	0.92–1.55	-	-
Employment status (ref: Employed)							
	Retired	443	43.8	0.93	0.73–1.18	-	
	Student/Apprentice	41	4.1	0.98	0.54–1.77	-	
	Unemployed/Inactive	116	11.5	1.32	0.91–1.92	-	
Educational level (ref: Obligatory schooling or less)							
	Vocational training	548	54.2	**0.68**	**0.49–0.92**	**0.72**	**0.52–0.99**
	Higher education	283	28	0.64	0.45–0.90	0.77	0.53–1.10
Lifestyle (ref: Couple (children)/With parents))							
	Couple (no children)	427	41.4	1.03	0.78–1.36	-	-
	Living alone with children	46	4.5	1.46	0.84–2.55	-	-
	Living alone	281	27.3	1.13	0.83–1.53	-	-
Perceived health score (scale from 0 to 100, continuous variable)							
		1,029	100	**0.99**	**0.98–0.99**	**0.99**	**0.98–0.99**
Body Mass Index (ref: Underweight or normal (<25 kg/m^2^))							
	Overweight (>25<30 kg/m^2^)	283	31	1.19	0.91–1.57	-	-
	Obese (>30 kg/m^2^)	211	23.1	1.15	0.85–1.56	-	-
Self-reported psychiatric diagnosis (ref: No)							
	Yes	27	2.6	**2.49**	**1.27–4.88**	-	-
Psychotropic treatment (ref: No)							
	Yes	64	6.2	**1.91**	**1.21–3.01**	-	-
Consultations with a GP in the last 12 months (ref: 0)							
	1–2	280	27	1.21	0.74–1.97	1.13	0.69–1.86
	3–5	320	31	1.48	0.91–2.42	1.42	0.87–2.34
	>6	359	34.9	**1.89**	**1.16–3.07**	**1.69**	**1.03–2.78**
Tobacco use (ref: No)							
	Yes	218	21.1	1.28	0.97–1.68	-	-
Hazardous alcohol consumption (audit-c) (ref: No)							
	Yes	366	35.5	1.08	0.86–1.36	-	-
Cannabis use (last 30 days) (ref: No)							
	Yes	47	4.6	1.36	0.79–2.35	-	-
Sports frequency (ref: Very often (>1/week))							
	Often (1/week)	195	18.9	1.05	0.77–1.44	-	-
	Occasionally	202	19.6	1.11	0.81–1.52	-	-
	Never	284	27.5	0.92	0.69–1.23	-	-
Balanced diet (ref: No)							
	Yes	887	86	**0.70**	**0.51–0.96**	-	-

Results in bold are significant.

## Discussion

The present study’s findings underlined the presence of significant mental distress among a primary care patient population: 27.6% were often (1/week) or very often (>1/week) stressed, 7.7% had a moderate-to-high PHQ-4 score and 53% were touched by at least one of the four sleep disorder criterias. Nevertheless, only 2.4% of participants reported being diagnosed with a psychiatric disorder. The PHQ-4 is a validated, widely-used screening tool, and a high score frequently leads to a diagnosis of depression or anxiety [19, 25]. However, we observed that these self-reported diagnoses were less frequent than reported mental health problems would suggest. In comparison, we can cite the OBSAN report about mental health in Switzerland, in which we see that in 2017, 15.1% of Switzerland’s population reported a moderate-to-high psychological distress [3]. Our finding could signal that mental health problems are under-diagnosed and may explain some of the under-treatment that we observed. In the general population of Switzerland, according to the OBSAN report already cited, during the 12 months preceding the survey of 2017, 5.4% of the participants had been diagnosed with a depression [3]. Concerning anxiety disorders, the global 12 months prevalence is 7.3%, which is way more than the reported diagnoses in our study [4].

Even if a pharmacological treatment is not always needed, we can observe that two thirds (67.9%) of patients with a self-reported psychiatric diagnosis were not being treated pharmacologically, and patients with a self-reported psychiatric diagnosis and/or undergoing psychotropic treatment were still very much affected by psychological suffering. For example, 26% of people treated with a psychotropic drug had a moderate-to-high PHQ-4 score. Our results showed that GPs need to better integrate mental healthcare into their daily practice, for example, by using screening scores to detect mental health problems or by integrating closer monitoring after initiating a psychotropic drug to evaluate symptom evolution and consequently adapt the medication. These findings should also encourage GPs and other stakeholders in the healthcare system to reflect more broadly on mental health and imagine new ways to improve primary care monitoring and follow-up for patients with mental health problems.

We found common determinants for the mental health problems among our specific sample and among the general population: poor mental health was associated with female sex, a low educational level (associated with sleep disorders in our study), and unemployment (associated with PHQ-4 in our study) [17, 18]. These three social indicators were also associated with high PHQ-4 scores in other studies, e.g., the German and Colombian general populations [27, 35]. For example, unemployment’s associations with poorer physical and mental health have been well described, due to, among other things, financial stress and the lack of the social status awarded to people in work [19, 36]. It should be noted, however, that people affected by such social determinants often feel oppressed, and it may be this that makes them vulnerable in terms of mental health rather than being unemployed *per se* [18].

The finding that female sex was associated with a higher frequency of stress and more sleep disorders was consistent with the existing literature. Various hypotheses have been put forward about this difference, such as women’s tendency to report their psychological symptoms more easily than men. The medicalization of women’s mental health could also lead to over-diagnosis among them and, conversely, under-diagnosis among men [20]. Moreover, diagnostic and screening methods may not necessarily have been adapted to women, and some sex differences have been seen to depend on the scales used [21]. Regarding depression–even if female sex is not associated with PHQ-4 score in our study - we can cite a recent study who revealed a new genetic clue to the higher rates of depression among women: a specific, non-coding RNA strand that can be upregulated in some women suffering from depression [37]. Links between mental health and sex need to be investigated further.

We noted an association between patients with more symptoms of depression and/or anxiety (higher PHQ-4 scores) and more sleep disorders tended to have consulted their GP more frequently in the last 12 months. One of the reason for this, is that many of these patients have a chronic or somatic disorder, and it is known that there is a high comorbidity between these and mental health problems [38]. GPs should, therefore, deliberately explore potential mental health issues among patients who consult very frequently, regardless of their reason for doing so. In addition, the active detection of mental health problems can be a valuable part of suicide risk prevention [39, 40]. We could also consider these results from another angle: close medical follow-up—as revealed by high numbers of consultations with a GP—is necessary for patients with mental health problems. In this context, GPs have a central role to play in creating robust physician–patient relationships and providing therapeutic support [41, 42].

Managing patients with mental health problems is a major challenge for GPs, and knowing when to refer patients to a mental health specialist is part of this task [43]. GPs must, therefore, be conscious of their professional and personal competencies and limitations, defining which situations require the intervention of a psychologist, a psychiatrist, or another mental health specialist. In Switzerland, an internship in a psychiatric hospital or institution is not mandatory for GP residents. Future GPs should be encouraged to do some clinical psychiatry before opening their practices, in order to acquire specific psychiatric skills, better refer patients to mental health specialists, and better collaborate with them [44]. In addition, prevention and public health are rarely addressed during medical school in Switzerland, as it is mainly focused on individual medicine. To develop a more global or community vision of health, future physicians should be made more aware of these subjects. In parallel, it would be interesting to explore the possibilities for interprofessional collaboration between GPs and mental health specialists. In other countries, some GPs work directly with mental health specialists in the same practice [45, 46]. In Switzerland, having a psychiatrist in a group practice has been tested, and the use of such practice configurations needs to be expanded [47].

Finally, we should not forget the GP’s role as a health promoter [48]. The present study found associations between physical activity, a balanced diet, and the absence of tobacco use and better mental health—associations that have been well demonstrated in previous studies [22, 49]. Some GPs prescribe walks or other physical activities in nature, and this has been shown to have an impact on mental health, especially when associated with contact with biodiversity [50, 51]. Taking the time for physical and mental health prevention activities during consultations is a major role of GPs in primary care [23, 24]. Screening tools such as the PHQ-4 can easily be used in this context, and other scores could be developed specifically for GPs. For example, without a validated tool to identify sleep disorders, a GP could use the four screening questions used in the present study.

### Strengths and Limitations

One of our study’s strengths is that it was carried out within a specific population monitored by GPs: the prevalence of mental health problems is usually studied within the general population. As GPs play a central role in treating mental health, conducting studies about this specific population seems important. Regarding patients with a self-reported psychiatric diagnosis or undergoing psychotropic treatment, we remained unaware of whether they were benefitting from psychiatric or psychotherapeutic monitoring or whether their psychotropic treatment was prescribed by a GP or another healthcare professional. It would be interesting to explore the links and types of collaboration between GPs and psychiatrists and other mental health specialists in primary care settings, to help us better understand how to optimize those collaborations and in which situations patients are referred to a specialist [52]. For example, GPs in Italy are more likely to refer their patients to a mental health specialist than GPs in France [12].

The present study’s results were based on a few questions selected from a much larger preventive healthcare survey, thus limiting the depth of our investigation into each subtopic. Future primary care studies should ask additional specific questions about mental health together with more in-depth investigations from a qualitative perspective.

The existence of a recall bias should be mentioned. Patients tend to remember their treatments more easily than their diagnoses. In addition, some psychotropic treatments can be given without a medical diagnosis. These two elements could explain some of the difference between the number of psychotropic treatments and self-reported psychiatric diagnoses recorded.

With regard to the score used to detect sleep disorders, we considered that one positive response or more was already considered a sleep disorder. This runs the risk of overestimating the sleep disorders actually present in patients.

Another important point is that we had no access to data related to non-pharmacological treatments. This limits any interpretation of the relationship between a psychotropic treatment and a participant’s symptomatology. Indeed, a pharmacological treatment alone is often insufficient, in many cases, it should be accompanied by psychotherapy. And in many situations, especially when the mental health problem is mild, the patient is sufficiently helped with psychotherapy or counseling, and pharmacological treatment is thus not always needed.

In the same idea, we did not have any data about the social support the patients had in their life. Indeed, a lot of studies have demonstrated the significant impact of social support on mental health, so it would be interesting to have this kind of information for more complete analyzes [18, 19].

The fact that the data utilized in this study were collected in 2015–16 is a limitation. The results do not take account of recent developments in the field. It’s also worth noting that the COVID-19 epidemic changed a lot in terms of mental health too, and if we were to redo the data collection today, the results would probably not be the same [53].

Finally, the study’s transversal design meant that we could not establish any causal links, only associations, thus limiting any interpretations of our results.

### Conclusion

Our study showed that a significant number of general practitioners’ (GPs) patients experience psychological symptoms and that specific social determinants are strongly associated with poor mental health. By identifying these determinants, GPs could monitor their patients more closely and better target screening. Indeed, mental health problems tend to be under-diagnosed in primary care settings. To provide patients with better care, GPs need to have a greater understanding of the distress caused by mental health problems and have the effective tools to manage them.

It is also important to know which patients to refer to a mental health professional and when their mental disorder may go beyond the usual scope of a GP’s skills. Better interprofessional collaboration in primary mental healthcare in Switzerland needs to be developed and implemented in order to improve overall healthcare provision.

GPs play a crucial role in their patients’ mental health. It is an integral part of their profession, in terms of prevention, treatment, and follow-up. Overall, mental health services must be better integrated into primary healthcare provision in order to provide patients with more comprehensive care.
